# Editorial: The Role of Primary Motor Cortex as a Marker and Modulator of Pain Control and Emotional-Affective Processing

**DOI:** 10.3389/fnhum.2017.00270

**Published:** 2017-05-23

**Authors:** Jorge Leite, Sandra Carvalho, Linamara R. Battistella, Wolnei Caumo, Felipe Fregni

**Affiliations:** ^1^Spaulding Neuromodulation Center, Spaulding Rehabilitation Hospital, Harvard Medical SchoolBoston, MA, United States; ^2^Neuropsychophysiology Laboratory, Psychology Research Center (CIPsi), School of Psychology (EPsi), University of MinhoBraga, Portugal; ^3^Portucalense Institute for Human Development (INPP), Universidade PortucalensePorto, Portugal; ^4^Physical and Rehabilitation Medicine Institute, Medical School General Hospital, University of São PauloSão Paulo, Brazil; ^5^Post-Graduate Program in Medical Sciences, School of Medicine, Universidade Federal do Rio Grande do SulPorto Alegre, Brazil; ^6^Laboratory of Pain and Neuromodulation, Universidade Federal do Rio Grande do SulPorto Alegre, Brazil

**Keywords:** motor cortex, stimulation, pain, cognition, emotion

In the 1940–50's Wilder Penfield and colleagues applied cortical electrical stimulation to patients undergoing epilepsy surgery to define what has become one of the landmarks on neuroscience: a map of the anatomical divisions of the body, divided in two cortical homunculi: sensory and motor (Penfield and Boldrey, 1937).

Ever since, the development of new tools to investigate brain function non-invasively increased knowledge about the structure and functions of the primary motor Cortex (M1) beyond motor control in both humans and animals. For instance, the role of M1 in visuomotor transformations, mental imagery, or mental rotation has been shown in studies dating more than 30 years ago (Georgopoulos and Pellizzer, 1995; Kosslyn et al., 1998). Also, M1 seems to be activated during memory retrieval of sensory information or finger tapping sequences after a short delay (Kaas et al., 2007), suggesting the M1 involvement with memory processes; as well as involved in language processing of action related words (de Lafuente and Romo, 2004; Hauk et al., 2004; Pulvermuller, 2005 for review). Furthermore, the involvement of the M1 region in higher cognitive functions has also been demonstrated in emotional processing. There seems to be a correlation between sensorimotor activation and empathy (Lamm et al., 2007), as well as relationship between sensorimotor activation and emotional processing in silent reading of emotionally laden words (Papeo et al., 2012). Moreover, M1 seems to be asymmetrically modulated by here emotionally laden sounds, with unpleasant sounds resulting in higher facilitation od motor evoked potentials in the left hemisphere, whereas pleasant sounds resulted in higher excitability in the right side (Komeilipoor et al., 2013).

The involvement of the M1 region in higher cognitive functions was also supported by a recent meta-analysis of neuroimaging findings in which an activation likelihood estimation was used to determine topographic convergence (Tomasino and Gremese, 2016). In the meta-analysis, the M1 subregion 4a was commonly activated during motor imagery and working memory, emotion/empathy, and language. But the potential role of M1 in higher cognitive functions is not limited to the activation of specific brain regions during task performance. By understanding how M1 modulates distant neural structures and its relationship with respective brain behavior, M1 can also be used as a potential marker for clinical applications, as well as to guide neuromodulatory therapeutic options (DaSilva et al., 2012; Carvalho et al., 2015). It is well known, for instance, that M1 has connections with several areas of the brain, and the stimulation of the motor cortex can induce changes in other systems (e.g., pain: Fregni et al., 2006; Castillo-Saavedra et al., 2016). Moreover, stimulation of motor cortex may actually improve cognitive functioning by the activation of cortico–striatal–thalamo–cortical loops (CSTC) (Leite et al., 2011).

Considering the role of M1 in cognitive functioning that surpass the motor processing, we proposed a research topic about the relationship between M1 and behavior, namely those related to pain and emotional-affective processing. We were interested in both theoretical and empirical contributions related to electrophysiological, pharmacological, neuroimaging, and neuromodulatory studies.

This special topic comprises 15 articles from a diverse group of scientists that provide a robust contribution for the development to the field. We also want to acknowledge the invaluable help that all reviewers provided during this process—many of them leaders in their field—whose contribution improved significantly the manuscripts. The reviews in this special issue investigate the role of motor cortex when using stimulation techniques to M1 to investigate pain modulation (Brasil-Neto) and how noninvasive brain stimulation can be used for reverting abnormal neuroplasticity associated with chronic pain (Naro et al.). This focus of M1 neuromodulation on pain modulation is also the focus of original studies in different types of pain, such as chronic musculoskeletal and post stroke pain, pain related to chemotherapy, fibromyalgia, or neuropathic pain (Botelho et al.; Caumo et al.; Hu et al.; Luu et al.; Mendonca et al.; O'Brien et al.). Additionally, a framework addressing the contralateral inhibition of the impaired hemisphere following stroke and its potential relationship with central post stroke pain is proposed (Morishita and Inoue). A second common theme was the use of EEG to understand changes in M1, and correlate this neural signal with pain and emotional processing in stroke patients (Doruk et al.) and chronic pain secondary to rheumatoid arthritis (Meneses et al.). Furthermore, the use of neuroimaging was also the topic of one study assessing connectivity alterations in patients with rheumatoid arthritis and correlating increased pain perception with increased connectivity for the supplementary motor areas, mid-cingulate cortex, and the primary sensorimotor cortex (Flodin et al.). Finally roles of the motor cortex on other cognitive domains were also explored, namely M1 activation with real or mental imagery (Galdo-Alvarez et al.), kinematic changes associated with pain in patients with fibromyalgia (Costa et al.), or changes in motor cortex activity following observation of emotionally laden pictures (Nogueira-Campos et al.).

This special topic highlights the role of the motor cortex that goes way beyond motor functioning. Also that we need to expand our knowledge about this particular region, its cortico–cortico and cortico–subcortico interactions, and how it can modulate or be modulated by different bottom-up (such as median nerve stimulation) or top down (such as TMS or tDCS) interventions. Despite that, this special topic clearly emphasizes methods to probe and neuromodulate motor cortex functioning and its potential impact for comprehensive rehabilitation (such as pain). But those are only a few examples of how motor cortex is involved in pain processing and higher order cognitive processing.

## Author contributions

All authors listed, have made substantial, direct and intellectual contribution to the work, and approved it for publication.

## Funding

JL and SC are supported by the Portuguese Foundation for Science and Technology (FCT) and European Union (FSE-POPH) with individual awards FRH/BPD/86027/2012) and (IF/00091/2015). JL, SC are members of CIPSi, supported by the Portuguese Foundation for Science and Technology and the Portuguese Ministry of Science, Technology and Higher Education through national funds and co-financed by FEDER through COMPETE2020 under the PT2020 Partnership Agreement (POCI-01-0145-FEDER-007653); and also through the Portuguese Foundation for Science and Technology PTDC/MHC-PCN/3950/2014. FF is funded by the following NIH grants: R21HD079048, R01HD082302, 1R44NS080632-01, 1R44AT008637, HD069776.

### Conflict of interest statement

The authors declare that the research was conducted in the absence of any commercial or financial relationships that could be construed as a potential conflict of interest.
